# Non-autonomous consequences of cell death and other perks of being metazoan

**DOI:** 10.3934/genet.2015.1.54#sthash.dNy9tFhS.dpuf

**Published:** 2015-02-05

**Authors:** Tin Tin Su

**Affiliations:** Department of Molecular, Cellular and Developmental Biology, 347 UCB, University of Colorado, Boulder, CO 80309-0347, USA

**Keywords:** *Drosophila*, imaginal discs, apoptosis, caspase, cancer

## Abstract

*Drosophila melanogaster* remains a foremost genetic model to study basic cell biological processes in the context of multi-cellular development. In such context, the behavior of one cell can influence another. Non-autonomous signaling among cells occurs throughout metazoan development and disease, and is too vast to be covered by a single review. I will focus here on non-autonomous signaling events that occur in response to cell death in the larval epithelia and affect the life-death decision of surviving cells. I will summarize the use of *Drosophila* to study cell death-induced proliferation, apoptosis-induced apoptosis, and apoptosis-induced survival signaling. Key insights from *Drosophila* will be discussed in the context of analogous processes in mammalian development and cancer biology.

## 1. Introduction

**Figure 1:**
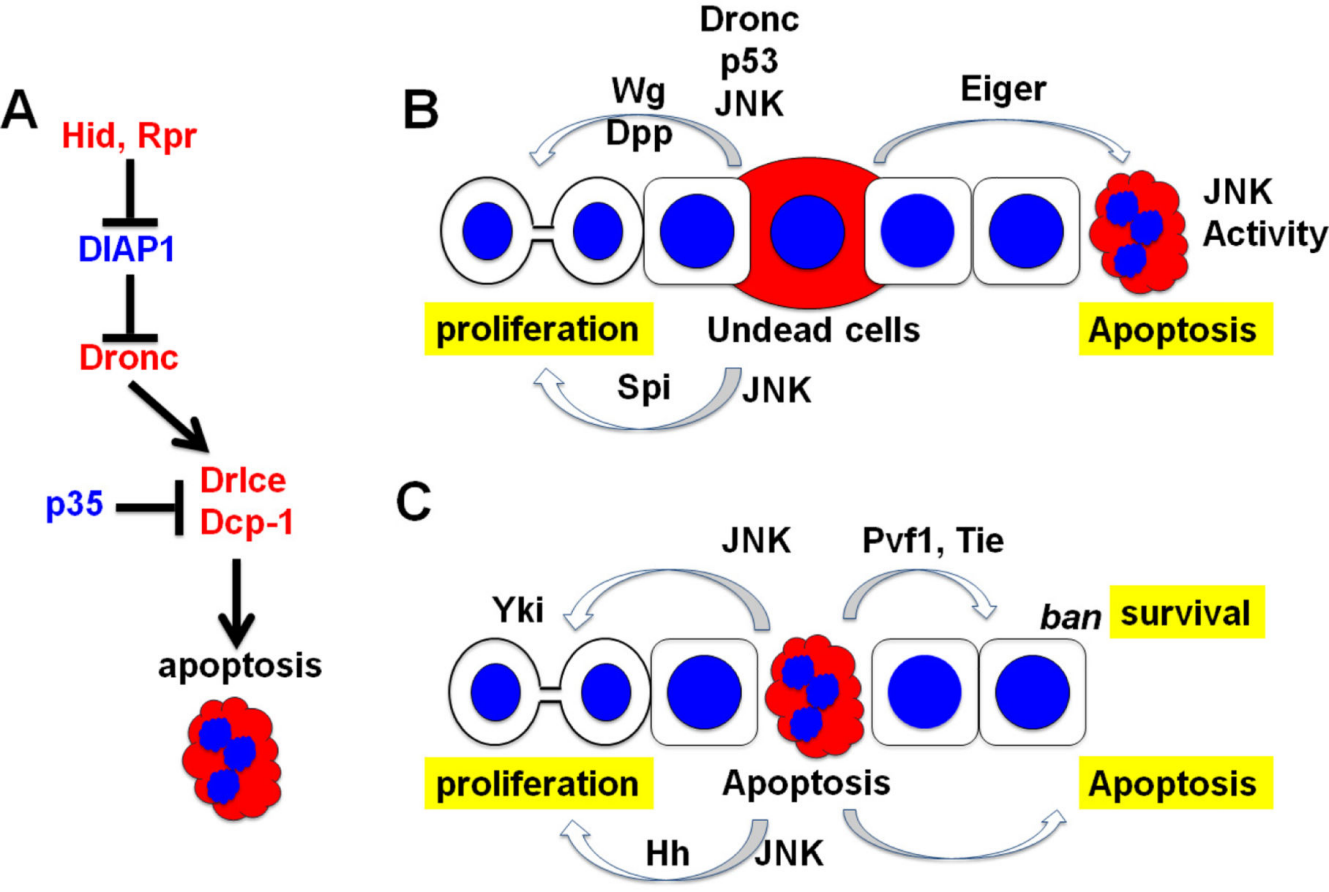
(A) *Drosophila* cell death pathway. Only the proteins discussed in this review are shown. Pro-apoptotic proteins are in red and anti-apoptotic proteins are in blue. Initiator caspase Dronc cleave to activate effector caspases Drice and Dcp-1. Their activity is kept in check by DIAP1. In response to apoptotic stimuli, Hid and Rpr overcome the effect of DIAP1 to induce apoptosis. p35 does not interfere with caspase cleavage but inhibits their activity. Dronc is refractory to inhibition by p35. (B) Undead cell-induced proliferation (left) and apoptosis (right). Co-expression of p35 with pro-apoptotic proteins generates undead cells that have initiated apoptosis but cannot complete it. These induce non-autonomous proliferation through Wg, Dpp and Spi, and apoptosis through JNK activation. Wg induction by undead cells requires Dronc, JNK and p53 in undead cells. Spi induction by undead cells requires JNK in undead cells. JNK activation to induce non-autonomous apoptosis requires *eiger* in undead cells. (C) Genuine apoptosis-induced proliferation (left) and survival/apoptotic signaling (right). The proliferative response in the wing disc is mediated by JNK activity in the apoptotic cells that activates Yki in the nearby cells. The proliferative response in the differentiating regions of the eye disc is mediated by Hh. Survival signaling occurs by activation of *ban* miRNA in the protected cells, which is dependent on effector caspase activity, Tie and Pvf1. Apoptotic cells also induce non-autonomous apoptosis but the molecular basis for this response remains to be understood.

Even after half of the cells in larval organ precursors have been killed by drugs or radiation, *Drosophila* larvae can emerge as adults that look indistinguishable from un-treated controls [1,2,3]. It is inferred that additional cell proliferation must have occurred to compensate for the cells lost to cell death, hence the term “compensatory” proliferation. Is compensatory proliferation an active response in which dying/dead cells signal to survivors to proliferate? Or is it simply filling in “gaps” left behind by dying cells, in a process akin to normal developmental controls that operate during organogenesis to achieve a seamless continuum of cells with appropriate identities? The scientific literature addressing these questions is complicated by the fact that different studies used different methods of killing cells, which we now know can produce different consequences. Further, in many studies, cells were induced to die but then kept alive by the co-expression of a caspase inhibitor, p35. This is possible because in *Drosophila*, the initiator caspase, Dronc (*Drosophila* NEDD2-like caspase), is mostly refractory to p35 whereas effector caspases, Drice and Dcp1 in *Drosophila*, are inhibited by p35 [4] (Figure 1A). Therefore, undead cells are in a suspended state with active Dronc but little or no Drice/Dcp1 activity. They have initiated apoptosis but have not completed it.

Signals from the so-called “undead” cells were thought to be similar to those from genuine dying/dead cells, only stronger and more durable. We know now that there are not only quantitative but also qualitative differences between the consequences of dying versus undead cells. To clarify the situation, it has been proposed that proliferation that restores organ size after tissue injury, for example by irradiation, be called “compensatory proliferation” and the proliferation that occurs in response to undead cells or genuine apoptotic cells, “apoptosis-induced proliferation” [5]. But the latter term can add to the confusion because undead cells are clearly not apoptotic. They have intact nuclei and cell membranes. They persist for days [6], and may even contribute to adult structures when induced in the larvae [7,8]. Therefore, this review will refer to what they cause as “undead cell-induced proliferation” and will distinguish it from the consequences of genuine apoptotic cells (Figure 1B & C).

Despite its name, the “undead” state may be physiological. p35 is encoded by baculovirus that naturally infects insect cells. The virus employs p35 to keep the host cell alive so that the virus can reproduce. Indeed, keeping host cells alive and proliferative is a common survival strategy utilized by many viruses including those with human hosts [9]. In many of these instances, host cell's apoptotic program is undermined by directed inhibition of pro-apoptotic pathways. So, in the arms race between the host and the virus, the state of an infected cell may very well resemble undead cells kept alive by p35 in *Drosophila*.

A word also about hormesis would be appropriate in the Introduction. Hormesis is a phenomenon in which low doses of an agent protects a subject from high doses of the same agent [10]. For example, mice previously exposed to low non-lethal doses of radiation were more resistant to sub-lethal doses of radiation compared to naïve mice [11]. Such examples of hormesis contrast with the consequences of cell death, the subject of this review; in the studies discussed here, the first treatment cells received was strong enough to cause their death. A version of hormesis that does involve initial cell death was hypothesized to cause “cell replacement repair” [12]. Here, the death of more sensitive cells, upon irradiation of a mouse for example, was proposed to stimulate surviving pluripotent cells to proliferate and replace the former. Studies in *Drosophila* provide experimental support for this hypothesis and identify key players that mediate cell replacement as discussed in following sections.

## 2. Non-autonomous effects of dying cells on the proliferation of surviving cells

### 2.1. Undead cells induce proliferation of nearby cells

In the first set of molecular genetic studies I will discuss, cells were killed by genetic manipulations or by externally applied stress in wing imaginal discs of *Drosophila* larvae [7,8,13,14]. Wing imaginal discs are precursors of the adult wing and thoracic tissues and are composed of columnar epithelial cells. During larval stages, wing imaginal discs are composed of discrete compartments such as Anterior (A) and Posterior (P), for example. Cells in the A and P compartments are of different lineage and do not mix [15]. Distinct gene expression patterns in each compartment allow compartment-specific expression of transgenes or recombinases to generate homozygous mutant clones of cells.

Directing the loss of *Drosophila* Inhibitor of Apoptosis Protein 1 (DIAP1; Figure 1A) or ectopic expression of pro-apoptotic proteins Hid or Rpr to specific compartments of the wing imaginal discs resulted in caspase activation and apoptosis within that compartment [8,13]. Exposure to ionizing radiation or application of heat shock also resulted in caspase activation and apoptosis [7,14]. Co-expression of p35 under these conditions generated undead cells. Induction of undead cells in isolated, marked clones resulted in increased proliferation of their cellular neighbors as seen by increased number of cells that incorporated BrdU, a marker for S phase, or were stained with an antibody of phosphorylated S10 on Histone H3 or pH3, a marker for mitosis [7,8]. Induction of undead cells in the P compartment of larval wing discs resulted in increased proliferation throughout the P compartment and also among cells of the A compartment that abut the P compartment. Thus, undead cells promote proliferation of the neighbors and this effect can cross the A/P compartment boundary. In discs in which undead cells have been induced throughout the P compartment, this compartment also showed rampant overgrowth, that is, increase in size beyond the normal range [7,8]. Overgrowth displayed both extra cells and abnormal cell arrangements into multiple layers.

### 2.2. The role of JNK, Wg and Dpp in undead cell-induced proliferation

Some of the undead cells in above-described studies exhibited elevated Dronc and Wg protein levels, and increased expression of a Dpp transcriptional reporter [7,8]. Wg (*Drosophila* Wnt) and Dpp (*Drosophila* TGF-β) are mitogens that could induce proliferation. Disruption of Wg signaling, by expressing a dominant negative version of TCF, a transcription factor and an effector in the Wg signaling pathway, throughout the P compartment abolished the mitogenic effect of undead cells within this compartment [8]. A functional role for Dpp in this context was not addressed. A later study used undead cells generated by the irradiation of p35 expressing cells, which also produced overgrowth. Wg and Dpp were shown to be required for overgrowth in this context, and for the induction of each other's expression [16].

Undead cells also showed elevated activity of Jun N-terminal Kinase (JNK) as detected by a transcriptional reporter for *puckered*, a downstream target of JNK. JNK is a MAP kinase family member with a conserved role in stress response. Puckered is a *Drosophila* JNK-specific MAPK phosphatase, and a feedback antagonist of JNK [17]. Interfering with JNK signaling in undead cells prevented the induction of Wg and inhibited the overgrowth [8]. Ectopic expression of Hep^AC^, a constitutively active form of a positive regulator of JNK, was sufficient to induce, Wg and Dpp expression, and overgrowth in the absence of p35 [8]. Indeed, Hep^AC^ was sufficient to induce Wg, Dpp, and overgrowth even in the absence of apoptosis in a *dronc* homozygous mutant background [16]. These findings led to the model that undead cells activate JNK, and it is JNK activity that mediates all other effects including the induction of Wg and Dpp mitogens and non-autonomous proliferation (Figure 1B).

Undead cells generated in two other types of larval epithelia also show elevated Wg and Dpp expression: antennae discs and parts of the eye imaginal disc in which cells are still proliferating and have not commenced differentiation [18]. Therefore, the induction of proliferation by undead cell through Wg may apply to many types of larval epithelia.

### 2.3. The role of Dronc and p53

Dronc is required for programmed cell death during normal development and for apoptosis induced by Hid or ionizing radiation [19]. p53 is also required for timely and efficient induction of apoptosis in response to genotoxic stress, although a reduced level of apoptosis can also occur with a delay in the absence of p53 [20]. Both Dronc and p53 are required for undead-cell induced proliferation. Moreover, this requirement may be distinct from their roles in the induction of apoptosis [8,13,19,21,22].

Initial studies using different dominant negative alleles of Dronc, expressed from different tissue specific promoters, produced contradictory results. Some studies found a role for Dronc in undead cell-induced proliferation (Dronc^C318S^ allele; [13]) while others did not (Dronc^C318A^ allele or the CARD domain of Dronc that lacks the catalytic region; [4,8]). Further analysis using classical loss-of-function alleles indicate a role for Dronc in undead cell-induced proliferation [22]. In this study, p53 was induced transcriptionally in a Dronc-dependent manner in the P compartment of wing imaginal discs when undead cells were generated in the same compartment. Importantly, both Dronc and p53 were required for the induction of Wg and for undead cell-induced overgrowth. Undead cells generated by ectopic co-expression of Hid and p35 in a *dronc^I29^* heterozygous null mutant background show cleaved caspase staining. Therefore, this level of Dronc was sufficient for caspase activation. But under the same conditions, undead cells could not induce overgrowth [22]. Therefore, the level of Dronc sufficient for caspase activation (by Hid) was too low for undead cell- induced proliferation, suggesting that contribution of Dronc to undead cell-induced proliferation occurs in addition to its contribution to caspase activation.

The role of p53 in promoting proliferation as noted in [22] is counter-intuitive to the known role of p53 homologs in cell cycle inhibition in response to checkpoints. The authors speculate that growth-promoting role of p53 reflects a more ancestral function of the p53 family that in vertebrates is fulfilled by the p63 homolog [22]. Additional work from the same authors supports the idea that p53 promotes growth and regeneration [21]; conditional expression of p53 was sufficient to promote regeneration responses that include Wg induction and tissue overgrowth. Moreover, these p53-dependent responses occurred in the absence of cell death, when tissue damage was induced by ionizing radiation in a *dronc* mutant background. These data are interpreted to mean that the role of p53 in regeneration, like that of Dronc, is separable from its role in damage-induced apoptosis. The finding that both isoforms of *Drosophila* p53 are capable of inducing apoptosis but only one of these is capable of promoting Wg induction and overgrowth provide further support for this idea [23]. A recent study extends the role of p53 in tissue regeneration to *Drosophila* germ line stem cells where p53 is needed for the recovery of stem cell proliferation after stress [24].

Given that Dronc, p53 and JNK are all required for Wg induction and overgrowth in response to undead cells, what is the relationship among Dronc, p53 and JNK? For apoptosis induction in response to stress, JNK and p53 function downstream of Dronc in a positive feedback loop to amplify apoptosis [25]. Such a positive feedback loop is in agreement with prior findings that Dronc is required for apoptosis but undead cells can also induce Dronc expression [26]. Similarly, inhibition of JNK using Puc blocked IR-induced apoptosis while a puc-lacZ, a transcriptional reporter for JNK, was induced after irradiation in wing discs with a similar schedule as induction of apoptosis under the same conditions [14,27]. Therefore, like Dronc, JNK promotes apoptosis but apoptosis also results in JNK activation. Ectopic induction of p53, in the absence of damage, caused apoptosis and activated JNK as seen by puc-lacZ reporter expression [14]; therefore, p53 could also participate in a positive feedback loop for apoptosis that includes Dronc and JNK. The question is whether JNK and p53 also function downstream of Dronc in a positive feedback loop to activate Wg in response to undead cells. p53 is known to function downstream of Dronc to induce Wg in response to undead cells [22]. As mentioned above, ectopic JNK activity, made possible by Hep^AC^, could induce Wg and overgrowth even in *dronc* mutants [16]; in other words, once active, JNK does not need Dronc to produce the effects of undead cells. But the initial activation of JNK may require Dronc in a non-apoptotic role.

### 2.4. Proliferation in response to undead cells in the eye disc

Generation of undead cells in the eye disc, using an *eyeless* driver to co-express *hid* and p35 also resulted in overgrowth [28]. In mosaic discs composed of undead and live cells, JNK was activated predominantly in undead cells and to a lesser extent in nearby live cells. A search for modifiers of overgrowth identified several upstream regulators of JNK as well as Spitz, an Epidermal Growth Factor (EGF) ortholog. Epistasis analysis placed Spi downstream of JNK, and EGFR pathway activation was seen in cells that did not overlap with cells showing active caspase staining in the eye discs. These results led the authors to propose that JNK activity in undead cells activates EGFR in nearby cells through Spi to promote overgrowth [28]. Additional work confirmed the role of EGFR/Ras/MAPK pathway in undead cell-induced proliferation in both eye and wing imaginal discs. Thus, Spi joins Wg and Dpp as mitogens generated by undead cells.

### 2.5. Proliferation in response to genuine dying cells

The mitogenic effect of Wg in response to undead cells raised the possibility that it had a role also in compensatory proliferation. Indeed, a large body of work on regeneration after surgical ablation indicate a central role for Wg [29]. In these experiments, parts of wing or leg imaginal discs were surgically removed and the remaining tissue cultured in vivo in the abdomen of adult females. Successful regeneration could occur after the removal of up to 25% of the disc. Regeneration post-surgery accompanies increased Wg expression and requires Wg. Consistent with these results, massive cell ablation using genetic means also resulted in regeneration that is dependent on Wg [30]. Here, Eiger, *Drosophila* TNF and a ligand for JNK, was induced in the wing pouch to result in substantial ablation of the pouch. The use of Rpr instead of Eiger also resulted in cell death but to a lesser extent. In both cases, remaining wing pouch cells showed induction of Wg and transcription factor Myc, and regenerated the pouch. RNAi-mediated depletion of Wg reduced the induction of Myc and other markers of regeneration such as cyclin E. Interestingly, Wg depletion had little effect on the final regenerated wing size, possibly due to incomplete removal of Wg by RNAi.

Given the role of Wg in undead cell induced proliferation and in regeneration after surgical or genetic tissue ablation, it came as a surprise that mutants of Wg are capable of regeneration after X-ray damage [16]. In these experiments, wing discs were generated in which the posterior compartments were comprised entirely of *dpp* or *wg* single mutant cells or *dpp wg* double mutant cells. Induction of cell death by X-rays, with and without p35, allowed the authors to assay for compensatory proliferation (without p35) and undead cell-induced proliferation (with p35) in the same context. *wg* and *dpp* mutants were capable of producing normal looking discs following irradiation to kill about half of the cells, but unable to induce overgrowth due to undead cells. In other words, undead cell-induced proliferation does require *wg* and *dpp*, in agreement with earlier studies. But compensatory proliferation that occurs in response to IR-induced apoptosis still occurred in the absence of *wg* or *dpp*, distinguishing compensatory proliferation from undead cell-induced proliferation. One caveat about these experiments is that Wg and Dpp are diffusible factors and even though the entire compartments were mutant for these genes, there still may be diffusion from the other compartment. Nonetheless, the finding that the same genetic manipulations with and without p35 produced different results still supports the idea that proliferation induced by undead and genuine dying cells has different requirements. This raises the possibility that additional factors contribute to proliferation in response to genuine dying cells. Molecules that can respond to disruptions in the epithelial cell layer would be good candidates.

### 2.6. Salvador/Warts/Hippo (SWH) tumor suppressor pathway

Wts and Hpo are serine/threonine kinases and Sav is an adaptor protein [31,32]. Upstream regulators of SWH include cytoskeletal regulators and cell adhesion molecules. A key downstream effector of SWH is the transcription factor Yorkie that is repressed by this pathway. Yki targets include proliferation and survival genes such as cyclin E and DIAP1. By killing cells in a variety of ways in marked clones in the wing imaginal disc but without the use of p35, the mitogenic effect of dying cells was found to reach 3 to 5 cells away [33,34]. In this context, SWH pathway was repressed and Yki activity was elevated in proliferating cells as seen by the transcriptional activation of Yki target genes [33]. Regeneration was sensitive to Yki gene dosage; Yki heterozygotes are unable to regenerate and adults emerge with wing defects when cell death was induced in the wing imaginal discs of the larvae. Thus Yki would fit into the role of molecules predicted to affect compensatory proliferation (Figure 1C). Interestingly, Yki^B5^/+ mutants used in these studies show no developmental defects. In other words, compensatory proliferation requires more Yki gene dosage than normal development. This argues against normal developmental mechanisms that operate during organogenesis operating also in compensatory proliferation. Finally, the effect of dying cells in these experiments did not cross the A/P compartment boundary, unlike the effect of undead cells as described above, further distinguishing the two responses.

What might be the signal that represses SWH pathway, activates Yki, and promotes compensatory proliferation? Likely candidates would be known upstream regulators of SWH that sense cell-cell contact such as atypical cadherins Dachsous and Fat and the FERM-domain protein Expanded [35]. Dachs is indeed needed, at least partially for the regeneration response; *dachs* mutants emerge as adults with small deformed wings when irradiated as larvae [33]. But *dachs*, *ft* and *ex* mutants still showed Yki activation in response to apoptosis, suggesting that other mechanisms mediate the latter. Concurrent studies implicate JNK in this process.

Mechanical wounding of wing discs is known to activate JNK [36], suggesting that JNK may also coordinate regeneration. Indeed, JNK has been identified as an activator of Yki in compensatory proliferation [34]. When cells in the wing imaginal discs were killed by ectopic expression of pro-apoptotic genes, puc-lacZ reporter for JNK activity was activated in dying cells and in cells adjacent to them. Reduction of JNK in dying cells interfered with Yki activation. Ectopic induction of JNK was sufficient to activate Yki even when apoptosis was reduced by Dronc mutations. Activation of SWH through overexpression of Wts or Hpo blocked the activation of Yki in this context. This could be because JNK acts through repression of SWH or because JNK acts in parallel to offset the repression of Yki by SWH. Regardless, these data place JNK as a key mediator of non-autonomous activation of Yki. In these experiments, in addition to Yki, Wg and Dpp were also induced in a JNK-dependent manner in response to dying cells. Further, activation of Yki and Wg followed different kinetics and activation of each was not dependent on the other [34]. Thus, these appear to be parallel outputs of JNK signaling. Because *wg* mutants can regenerate but Yki mutants cannot, activation of Yki by JNK appears to make a greater contribution to compensatory proliferation than activation of Wg by JNK.

We note that depletion of actin capping proteins by directing RNAi to specific compartments in wing imaginal discs resulted in cell autonomous apoptosis and JNK activation. Keeping such cells alive with p35 resulted in overgrowth within the same compartment, as well as a low level of increased proliferation outside the compartment [37]. Co-expression of a dominant negative JNK or depletion of Yki using RNAi in undead cells reduced the overgrowth, but indirect roles such as interference with the cell death program in undead cells has not been not ruled out. We note also that *patched* mutant clones in the eye imaginal discs undergo cell death [38]. Immortalization of *ptc* mutant cells using mutations in *Drosophila* Apaf1-related protein Ark resulted in non-autonomous activation of Yki and tissue overgrowth. It is unclear if immortalized cells in this case are undead or simply live cells with abnormal *Hh/ptc* signaling.

### 2.7. Proliferation in response to genuine apoptotic cells in the eye disc

Wing imaginal discs studied in the above-described reports were composed primarily of proliferating cells during larval stages. In contrast, in eye imaginal discs at the same larval stages studied, some of the cells have exited the cell cycle and begun to differentiate. Increased proliferation also occurs in response to experimentally induced apoptosis in the parts of the eye discs with differentiating cells using the GMR driver, as seen by elevated number of cells with BrdU incorporation and pH3 staining [18]. Cells that participated in extra proliferation showed characteristics of undifferentiated cells; it was speculated that these cells could represent post-mitotic cells that had begun differentiation but re-entered the cell cycle in response to apoptosis of their neighbors. The signaling molecule Hedgehog is normally expressed in the differentiating cells at this stage but increased Hh protein was observed in cells induced to die. Inhibition of Hh with a temperature sensitive allele of *hh* or by interfering with Hh signaling using mutants forms of downstream effectors Ci and *smoothened* blocked the extra proliferation. Neither Wg nor Dpp was induced under these conditions in the differentiating regions of the eye disc and p53 was not required. Thus proliferation in response to genuine apoptosis in the differentiating regions of the eye disc required Hh but not p53, Wg, or Dpp. Co-expression of p35 or mutations in effector caspases abolished this proliferation, indicating that the activity of effector caspases was required for proliferation in response to apoptosis in this tissue. Further, undead cells generated did not produce overgrowth as they did in the wing imaginal discs.

In another iteration of the experiment, transient expression of Hid in the dorsal half of the eye discs induced apoptosis in regions with and without differentiating cells [28]. Such discs showed increased JNK and EGFR activity and increased expression of EGFR ligand Spi, and regenerated to restore normal size and patterning of differentiating cells. Reduction of JNK or Spi through RNAi or heterozygosity interfered with regeneration, indicating that JNK and EGFR/Ras/MAPK, which mediate overgrowth in response to undead cells (see the section 2.4.), also participate in proliferation in response to genuine apoptosis [28]. It would be interesting to investigate whether and how the EGFR pathway integrates with the Hh pathway in responding to apoptotic cells in the eye disc.

We note that ligand-independent de-regulation of Hh, typically by mutations in the upstream regulators such as *ptc* and *cos2*, results in non-autonomous proliferation and survival signaling (for example, [39,40]). But because cells with deregulated Hh are not necessarily dying, these studies fall outside the scope of this review.

### 2.8. Method of killing matters

I mentioned briefly the role of Wg in regeneration after surgical removal of large sections of wing and leg discs [29]. Massive cell ablation to a similar degree but using genetic means killed cells in a defined domain that comprises about 20% of the wing disc and almost the entire wing pouch that will generate the wing proper [30,41]. These studies show that the exact pro-apoptotic gene used matters. Killing cells with Rpr or Eiger resulted in Wg induction but killing cells with Hid did not, at least in the wing disc [41]. The latter study using Hid found an initial systemic response of elevated mitotic activity throughout the disc, followed by a more localized proliferative response within the domain of death. Other responses included the migration of cells from outside to inside the death domain. Increased JNK activity was observed first at the periphery of the death domain and later throughout the death domain. Inhibition of JNK within the dying cells interfered with the regeneration responses whereas inhibition of JNK outside the death domain interfered with the migration of cells into the death domain and with regeneration [41]. In contrast, when Rpr was used instead of Hid in a similar experimental protocol, increased proliferation was observed initially in the vicinity of the dying cells and later in more distant regions of the disc [42]. In these studies, JNK was activated at the leading edge of cells outside the death domain but not in dying cells. Further, while mutations in JNK interfered with regeneration, inhibition of JNK specifically within the dying cells did not have an effect, suggesting the requirement for JNK is outside the death domain. Clearly more work will be needed to understand these differences.

### 2.9. Non-autonomous effect on proliferation; parallels in mammals

A response called “Phoenix Rising” occurs in mice after cell killing by ionizing radiation and is mediated by prostaglandins, molecules known to participate in localized autocrine or paracrine signaling. Here, the activity of Caspase 3 and 7 is required in dying cells and mediates the activation of phospholipase A_2_ and the subsequent production and release of prostaglandin E_2_, a stimulator of cell proliferation [43]. These signals act non-autonomously to stimulate proliferation and tissue regeneration. Follow-up studies using this system in vitro identified Sonic Hedgehog signaling as an important player [44]. Irradiated cancer cells showed increased expression of Shh and Gli1, a transcriptional activator and an effector of Shh signaling. The mitogenic effect of irradiated cancer cells was stimulated by Shh agonists and inhibited by Shh antagonists. Gli1 knockdown in irradiated dying cells compromised their mitogenic effect while Shh agonists alone were able to stimulate proliferation of the recipients in the absence of irradiated dying cells. Thus, Shh signaling appears to act in both the dying cells and the recipient cells for this effect. How Shh fits in with caspases and prostaglandins remains to be determined. Regardless, this phenomenon appears to be highly relevant to radiotherapy of cancers. Deficiency of caspase 3, either in xenografted tumors or in the stroma provided by caspase 3^−/−^ mice as host, caused substantial tumor sensitivity to radiotherapy [45]. These results can be explained by the earlier finding that dying cells release mitogenic factors in a caspase-dependent manner following irradiation. Such signals, either from the tumor or the stroma, would allow tumor regeneration following radiotherapy and hence resistance. Without caspase 3, irradiation may not produce a similar mitogenic outcome, hence the increased sensitivity of tumors to radiotherapy. This relationship may be applicable to human patients; the level of active Caspase 3 or even Caspase 3 mRNA pretreatment correlated with tumor recurrence after radio or radio-chemotherapy or relapse during therapy in human cancer patients [45].

The finding that the use of caspase 3^−/−^ mice as host increased the sensitivity of xenografted tumors to radiotherapy illustrates the contribution by the stroma [45]. The role of the stroma, and in particular the fibroblasts, in the proliferation and survival of cancer cells through WNT signaling has also been identified [46]. This study found that the expression of 48 genes that encode extracellular proteins were up-regulated in a prostrate fibroblast cell line after treatment with two common chemotherapy drugs, mitoxantrone, a topoisomerase II inhibitor, and a docetexal, an inhibitor of microtubule dynamics. WNT16B topped the list and was induced also in irradiated fibroblasts in vitro or in fibroblasts of patients after chemotherapy. Conditioned media from fibroblasts with constitutive WNT16B expression or conditioned media from irradiated fibroblasts were found to promote proliferation, survival and migration of epithelial cancer cells in vitro. Co-culture of cancer cells with WNT16B expressing fibroblasts or irradiated fibroblasts enhanced tumor growth in mouse xenograft models. These data indicate that fibroblasts exposed to radiation or chemotherapy agents secrete WNT16B that promotes the proliferation and survival of cancer cells. Whether caspase activity or apoptosis of fibroblasts were needed for this effect remains to be investigated.

An interesting difference between Shh mediated mitogenic effect of dying cells and WNT16B mediated mitogenic and pro-survival effect of fibroblasts was that the former was exhibited by both fibroblasts and cancer cells where as the latter effect was seen only in fibroblasts and not in benign or malignant epithelial cells. In a tumor comprised of both cancer cells and fibroblasts, it remains possible that both mechanisms operate to promote the survival and proliferation of cancer cells in response to prior damage. The fact that two key mediators of non-autonomous mitogenic effects in response to damage in mammals and *Drosophila* are Wnt/Wg and Hh/Shh homologs attest to the usefulness of *Drosophila* as a metazoan model.

## 3. Non-autonomous effect of dying cells on survival/death of neighbors

### 3.1. Apoptosis-induced apoptosis

When apoptosis was induced using ricin toxin in the posterior or the dorsal compartments of wing imaginal discs, apoptotic cells were observed in the anterior or the ventral compartment, respectively [3]. Similarly when undead cells were generated in the P compartment of wing imaginal discs using Hid and p35, apoptotic cells were observed in the A compartment [22]. These studies show that apoptotic and undead cells can induce non-autonomous apoptosis. The effect was robust if pro-apoptotic genes, Hid or Rpr in this case, were continuously expressed, but the effect was weaker if pro-apoptotic gene expression was limited to 72 hours [47]. Eiger was required in the undead cells and JNK was required in the secondary apoptotic cells for this effect (Figure 1B). Induction of apoptosis for 2–4 days in the absence of p35 was also able to induce non-autonomous apoptosis. The authors propose that this phenomenon may allow for coordinated removal of cells, by propagating cell death within a tissue. Such coordination would serve, for example, to sculpt tissues during organogenesis. Indeed, the authors found that apoptotic cells express TNF-α during synchronized bouts of developmental cell death that occur during the natural life cycle of hair follicles in mice [47]. Neutralization of TNF-α was found to disrupt and to de-synchronize the cycles of programmed cell death. Therefore, conserved TNF signaling mediates apoptosis/undead cell-induced apoptosis in both *Drosophila* and mice.

### 3.2. Apoptosis-induced survival signaling

We have found that when cells in the wing imaginal discs were killed using ionizing radiation, induction of pro-apoptotic genes or by depletion of essential proteins, the surviving cells became harder to kill by IR or a chemical microtubule depolymerizing agent [48]. The non-autonomous protective effect of dying cells was seen as little as 6 hours after the initial induction of primary apoptosis. The minimal requirements for the protective effect consisted of effector caspase activity in the dying cells, *tie* that encodes a receptor tyrosine kinase, and *bantam* miRNA (Figure 1C). *ban* was shown previously to repress *hid* expression and thereby provide an anti-apoptotic function [49]. Induction of a putative Tie ligand, Pvf1, was observed in dying cells using a transcriptional reporter while *ban* activation was seen in protected cells using a *hid*-3’UTR reporter. We proposed that dying cells signal through Pvf1 and Tie to activate *ban* in the neighbors and protect the latter from further loss. We have termed this effect the Mahakali effect, after a Hindu goddess of death who protects her followers. We do not believe the Mahakali is related to hormesis, a phenomenon in which a lower dose of an agent protects a subject against a higher dose of the same agent. In fact, we found that the higher the initial cell death, the stronger the Mahakali effect [48], which would be contrary to hormesis in which low initial doses produce a strong protective effect.

The Mahakali effect, similar to apoptosis- or undead cell-induced apoptosis, could cross the A/P compartment boundary. But regarding the outcome, the Mahakali effect by which dying cells make survivors more resistant to IR- and chemical-induced apoptosis appears to be the opposite of apoptosis/undead cell-induced apoptosis. But there may be a way to reconcile these differences. Induction of apoptosis by dying/ undead cells was observed 2–4 days after the initial induction of dying/undead cells. Mahakali effect was detectable as little as 6 hours after cell death induction using similar approaches (Hid/Rpr). It is possible that apoptosis confers a fast anti-apoptotic response that is followed by a slow pro-apoptotic response in the survivors.

### 3.3. Non-autonomous effects on survival in mammals

In the classical “radiation bystander effect”, the effect of irradiated cells on the neighbors is destructive, making the latter more prone to death by cytotoxic agents. Bystander effect has been described in mammalian cell culture and in mice [50,51,52,53]. There is evidence that the signal is soluble; media from irradiated cells can induce bystander effect on naïve cells. Inhibitors of bystander effect include antioxidants L-deprenyl and lactate [52], suggesting that oxidative stress and energy metabolism may be involved in radiation bystander effect. The Mahakali effect in which the outcome is pro-survival would be the opposite of the radiation bystander effect. Instead, the pro-survival signaling by irradiated fibroblasts through WNT16B signaling that is described in the preceding section would be similar in spirit to the Mahakali effect although the latter operates through Tie. Interestingly, in the study that identified WNT16B as a mediator of non-autonomous mitogenic and survival signaling, among other extracellular proteins up-regulated by fibroblasts treated with chemotherapy agents was ANGPT1, a known ligand for mammalian Tie-2 [46]. It would be interesting to investigate whether Tie homologs also participate in non-autonomous protective effects of stressed cells in mammals.

## 4. Questions for the future

In summary, dying and undead cells can exert profound effects on proliferation and death of surviving cells. JNK signaling has emerged as a key mediator of most aspects of these effects, functioning not only in the dying/undead cells but also in the recipient cells as in the case of undead-cell induced apoptosis (Figure 1B & C). In addition, non-canonical roles for p53 and initiator caspase Dronc have emerged the case of undead cell-induced proliferation (Figure 1B). Soluble signals that mediate the effect of dying/undead cells include Wg, Spi and Pvf1. Yet, there remain several unresolved issues that pose questions for the future.

There is emerging evidence that influences of dying cells on their neighbors may show tissue specificity. For example, Yki activation in response to JNK activation was strong in wing and haltere discs but was reported to be weak or absent in leg and eye discs [34]. In another example, undead cell-induced apoptosis was observed in wing, leg and haltere discs but not in the eye-antennal discs [47]. In another example, ectopic induction of Hid resulted in induction of Wg in the eye disc [28], but not in the wing disc [41], again illustrating that different tissues respond differently to the same cell death inducers. Therefore, an important goal would be to understand the molecular basis for these differences.

Another aspect of these studies that requires further attention concerns the method of cell kill. A comparative analysis of the consequences of cell killing using Hid, Rpr, p53, Eiger, Hep^CA^ (constitutively active JNK pathway) or DIAP1^RNAi^ found clear differences in Wg activation and regeneration even when some of the methods produced similar levels of cell death [41]. Understanding how these quantitative differences translate into different non-autonomous consequences would be necessary to completely understand how cells behave in a multicellular context.

A component that is missing from many of the studies concerns the contribution from the immune system. *Drosophila* immune system is simpler than that of mammals and provides innate but not acquired immunity. There is evidence that macrophage-like cells travel to and engulf apoptotic or undead cells in situ [3,8]. Macrophages are known to produce high levels of Pvf1 and Pvf2. We have found that Pvf1 mutants are defective for the non-autonomous protective effect of dying cells [48]. This raises the question of whether macrophages recruited to the site of dead and dying cells produce Pvf1 and thereby contribute to Tie signaling events that result in increased survival of the neighbors.

The decision to live or die for a cell is governed by a balance between pro and anti-apoptotic functions. Therefore, a reduction in one may influence apoptosis by lowering the threshold for the opposing activities to overcome, thus serving a permissive role. On the other hand, a factor that becomes activated in response to dying or undead cells may overcome the threshold to induce apoptosis in the neighbor, thus serving an instructive role. Both types of contributions are, of course, important but instructive events indicate changes in response to dead and dying cells but permissive, threshold-setting effects reflect hard-wired relationships. Understanding instructive versus permissive role of contributing activities is an important goal.

Finally, there are other types of cell death besides apoptosis: necrosis, autophagic death and clonogenic death, which resembles senescence in that cells stop proliferating in both cases. What are their non-autonomous consequences? Senescence is known to promote non-autonomous tissue dysfunction [54]. It would be interesting to see if other types of cell death also produce non-autonomous signals that affect the live-death decisions of other cells. There is mounting evidence that non-autonomous signals generated by damaged tissues play a critical role in homeostasis at the organism level. For example, in mice, muscle injury at one site in the body is communicated to other sites for non-autonomous “priming” of muscle stem cells for proliferation [55]. Likewise, mitochondrial stress response that occurs due to the depletion of one member of multi-member enzymes in the neurons *C. elegans* can activate a mitochondrial stress response in the gut of the same animal. This tissue non-autonomous response serves a protective purpose and delays organismal aging [56]. Indeed, these and other non-autonomous effects discussed here by which stress in one part of the body is communicated to other parts of the body for the overall benefit of the organism may represent the essence of what it means to be a metazoan.

## 5. Conclusion

It is becoming increasing clear that, to fully understand diseases of cellular misbehavior such as cancer, one must pay attention to the “social” context of cells as in tumor-stroma interactions. *Drosophila* researchers have been doing precisely that for many decades, in deciphering normal and abnormal cell-cell interactions. Examples cited in this review illustrate how, even in death, cells can influence the behavior of their neighbors. Powerful genetics and molecular tools in *Drosophila* have made it possible to detect and study these effects, and will allow us to identify new non-autonomous consequences of dying cells. Application of this knowledge to vertebrate and mammalian systems should provide us with a better understanding of how cells live and die in a multicellular context and opportunities to exploit these mechanisms for improved treatment of disease.
